# The detection of zoonotic microorganisms in *Rhipicephalus sanguineus* (brown dog ticks) from Vietnam and the frequency of tick infestations in owned dogs

**DOI:** 10.3389/fvets.2024.1435441

**Published:** 2024-08-12

**Authors:** Thom Do, Linh Khanh Bui, Rika Umemiya-Shirafuji, Tawin Inpankaew, Tanjila Hasan, Iqra Zafar, Zhuowei Ma, Li Hang, Uday Kumar Mohanta, Moaz Amer, Shimaa Abd El-Salam El-Sayed, Xuenan Xuan, Ketsarin Kamyingkird

**Affiliations:** ^1^National Research Center for Protozoan Diseases, Obihiro University of Agriculture and Veterinary Medicine, Obihiro, Hokkaido, Japan; ^2^Department of Parasitology, Faculty of Veterinary Medicine, Vietnam National University of Agriculture, Hanoi, Vietnam; ^3^Department of Parasitology, Faculty of Veterinary Medicine, Kasetsart University, Bangkok, Thailand; ^4^Department of Biochemistry and Molecular Biology, Faculty of Veterinary Medicine, Mansoura University, Mansoura, Egypt

**Keywords:** dogs, ticks, tick-borne pathogens, risk factors, Vietnam

## Abstract

Dog owners are greatly concerned about tick infestations in their pets. The prevalence and dispersion of ticks and their disease-causing microorganisms have been limited from the viewpoint of dog owners in Vietnam. This study investigated the presence of tick infestation and the pathogens associated with it in canines that were brought to veterinary hospitals in Vietnam. In the survey, 1,423 dogs participated from February to October 2022. Molecular and morphological methods were utilized to identify ticks and the associated pathogens. In addition,risk variables linked to tick infestation were documented and analyzed using statistical methods. The total exposure to the brown dog tick (*Rhipicephalus sanguineus sensu lato*) was 29.01%. Nam Dinh has the highest tick prevalence among the research areas. Tick infestation reached its highest point between June and September in the northern region of the country, with distinct seasons showing a strong correlation with tick infestation in dogs. Out of 177 tick pools examined, 146(82.49%) tested positive for at least one infection. *Mycoplasma* spp. (78.53%) was the most common, followed by *Anaplasma* spp. (37.29%), *Rickettsia felis* (5.08%), *Babesia vogeli*, and *Hepatozoon canis* (2.82%). In the current study, there was a statistically significant link between tick infestation and characteristics such as age, breed, body size, lifestyle, and bathing frequency. Understanding the seasonal behavior of vector ticks is crucial for identifying individuals or animals susceptible to tick-borne diseases. Studying the distribution of ticks and their ability to carry and disseminate zoonotic germs in specific places could assist veterinarians and policymakers in implementing effective strategies to manage zoonotic infections.

## 1 Introduction

Ticks are significant blood-feeding carriers of disease-causing agents that impact both pets and humans worldwide. Ticks can transfer many zoonotic pathogens such as viruses (tick-borne encephalitis virus), bacteria (*Anaplasma, Ehrlichia, Rickettsia*), and protozoa (*Babesia* and *Hepatozoon*) to vertebrate hosts during blood feeding, which might damage their health (1). More than seven tick species have been recently found in dogs in Asia, such as *Rhipicephalus haemaphysaloides, Rhipicephalus sanguineus, Haemaphysalis longicornis, Haemaphysalis campanulata, Haemaphysalis wellingtoni, Haemaphysalis hystricis,* and species within the genus *Ixodes* (2). Among these, *R. sanguineus*, also known as the brown dog tick and taxonomically designated as *R. sanguineus sensu lato* (s.l.) of tropical lineage, was the predominant tick species infesting dogs in numerous nations (2). *Rhipicephalus sanguineus* is believed to transmit many infections that cause babesiosis, hepatozoonosis, ehrlichiosis, mycoplasmosis, and rickettsiosis in dogs throughout Southeast Asia (3, 4). Apicomplexan protozoa parasites often found in dogs worldwide include species of *Babesia* (*Babesia canis*, *Babesia gibsoni*, and *Babesia vogeli*) and species of *Hepatozoon* (*Hepatozoon canis* and *Hepatozoon americanum*) (5, 6). Anaplasmataceae bacteria, including *Ehrlichia canis* and *Anaplasma platys*, are the most common bacterial tick-borne diseases found in dogs in Asia. All these infections are transferred through the vector’s bite during blood feeding, except for *Hepatozoon*, which is transmitted through the ingestion of *R. sanguineus* (7).

Vietnam is one of the rapidly developing economies in Asia, leading to an increase in the number of companion animals. Vietnam is geographically organized into three primary regions: Northern, Central, and Southern. It features both tropical and temperate temperature zones. In contrast to the South, which has two different seasons—dry and rainy—the North experiences four distinct seasons: spring, summer, autumn, and winter. The northern regions experience summertime averages of 22°C–27.5°C and wintertime averages of 15°C–20°C. Southern regions see consistently high temperatures ranging from 26°C to 29°C year-round.^1^ So far, the only type of tick found on dogs during studies on ticks in dogs in Vietnam was the brown dog tick (2, 8, 9). Protozoa and bacterial tick-borne organisms were recently found in dogs in Vietnam, with a detection rate ranging from 0.8% to 25.8% (2). The presence of a large dog population, favorable weather conditions, and close contact between humans and pet dogs facilitate the spread of zoonotic tick-borne diseases in the area. There have been few surveillance efforts to quantify tick abundance in dogs and to comprehend their frequency and spatial distribution in Vietnam. This study aimed to survey ticks infesting privately owned dogs at veterinary hospitals in Vietnam and detection of selective tick-borne pathogens that are reported commonly in dogs and ticks in Asia. It involved morphological and genetic species characterization of the collected ticks. Factors contributing to tick infestation were documented and examined.

## 2 Materials and methods

### 2.1 Ethical consent

The Ethics Committee of Obihiro University of Agriculture and Veterinary Medicine accepted the protocol for using animal samples in this work (Permit for animal experiment: 21-25; DNA experiment: 1725-5).

### 2.2 Area of study, collection of samples, and tick identification

**Figure 1:**
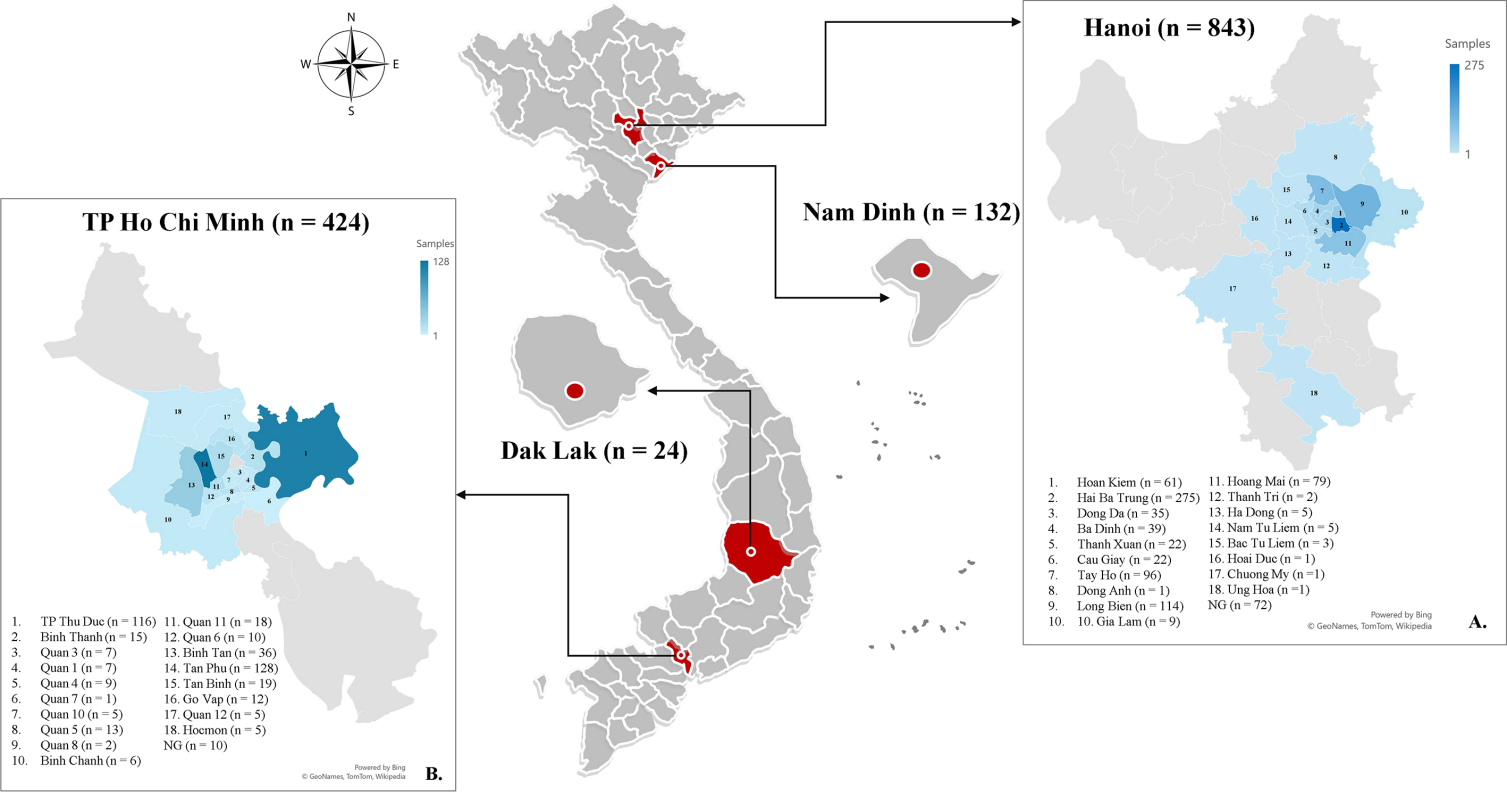
Map showing the locations where tick infestation on dogs is being studied in Vietnam, with red circles indicating the sampling sites in each province. The blue highlighted districts indicate the specific geographic areas where samples were gathered in Hanoi **(A)** and Ho Chi Minh City **(B)**.

A survey on tick infestation in dogs was conducted from February to October 2022 with voluntary participation from veterinary clinics in Hanoi (21°01′N, 105°51′E), Ho Chi Minh City (10°46’N; 106°42′E), Nam Dinh (20°25′N; 106°10′E), and Dak-Lak (12°4′N, 108°3′E) in Vietnam (Figure 1). Veterinarians conducted a comprehensive inspection of the animals to detect ticks by inspecting the entire body surface for more than 5 min. The examinations involved assessing the animals’ physical condition and noting any abnormalities in the skin/haircoat and mucous membranes such as jaundice, paleness, or hemorrhaging. A questionnaire was created to collect data about the sampled dogs’ location, breed, sex, age, and body size for analyzing several categories related to tick infestation. Further inquiries were made regarding the living environment (indoor, outdoor, or semi-outdoor) and bathing frequency (weekly, monthly, every 2 months, or annually) if applicable.

From one to five ticks attached to the dog were collected and stored in 1.5 mL tubes containing 70% ethanol. The specimens were subsequently transferred to the National Research Center for Protozoan Diseases in Obihiro, Hokkaido, Japan for identification using morphological and molecular analysis. A total of 555 ticks were submitted for species identification and detection of microorganisms. Ticks were examined using a stereomicroscope (Olympus SZX16) to determine their species and life stage (larva, nymph, or adult) by utilizing specific physical characteristics (10). Following the initial morphological identification, the ticks were rinsed extensively with phosphate-buffered saline. Afterwards, ticks in the identical life stage and obtained from the identical dog were pooled for the DNA extraction using an DNeasy Blood and Tissue Kits (Quiagen, Germany) as per the manufacturer’s guidelines. Following the pooling process, a total of 177 pools was obtained, with each pool containing between one and five individual ticks for DNA extraction. Following DNA extraction, the sequences aimed at the species-specific gene were amplified to molecularly confirm the tick species and their associated microorganism (Table 1).

**Table 1:** Primer sequences used for pathogen detection in ticks in this study.

Target pathogens (target gene)	Primer sequences (F/R) 5′-3′	Product size (bp)	Annealing temperature (°C)	References
Tick species (*Cox1*)	GGTCAACAAATCATAAAGATATTGG	710	55	(11)
	TAAACTTCAGGGTGACCAAAAAATCA			
*Rickettsia* spp. spotted fever group (*OmpA*)	GCTTTATTCACCACCTCAAC	212	55	(12)
	TR(G/A)A TCACCACCGTAAGTAAAT			
*Rickettsia felis* (*gltA*)	F1 GCAAGTATTGGTGAGGATGTAATC	654	58 & 55	(13)
	R1 CTGCGGCACGTGGGTCATAG			
	F2 GCGACATCGAGGATATGACAT			
	R2 GGAATATTCTCAGAACTACCG			
*Mycoplasma* spp. (*16S rRNA*)	ATACGGCCCATATTCCTACG	595–618	60	(14)
	TGCTCCACCACTTGTTCA			
Anaplasma/Ehrlichia (*16S rRNA*)	GGTACCTACAGAAGAAGTCC	345	52	(15)
	TAGCACTCATCGTTTACAGC			
*Anaplasma platys* (*groEL*)	AAGGCGAAAGAAGCAGTCTTA	724	54	(16)
	CATAGTCTGAAGTGGAGGAC			
*Ehrlichia canis* (*gltA*)	TTATCTGTTTATGTTATATAAGC	1,372	50	(17)
	CAGTACCTATGCATATCAATCC			
*Bartonella* spp. (*16S-23S rRNA*)	(C/T)CTTCGTTTCTCTTTCTTCA	260	55	(18)
	AACCAACTGAGCTACAAGCC			
Babesia/Hepatozoon (18S rRNA)	CCAGCAGCCGCGGTAATTC	350–400	57	(19)
	CTTTCGCAGTAGTTYGTCTTTAACAAATCT			
*Babesia canis* (*18S rRNA*)	GCW(A/T)TTTAGCGATGGACCATTCAAG	208	60	(20)
	CCTGTATTGTTATTTCTTGTCACTACCTC			
*Hepatozoon canis* (*18S rRNA*)	ATACATGAGCAAAATCTCAAC	666	57	(21)
	CTTATTATTCCATGCTGCA			

F, Forward; R, Reverse; Cox1, cytochrome c oxidase subunit I; ompA, outer membrane protein A; groEL, heat shock protein gene; gltA, citrate synthase gene.

### 2.3 Molecular detection and phylogenetic analysis of microorganism

The DNA samples underwent screening for apicomplexan protozoa (such as *Babesia* spp., *Hepatozoon* spp.), bacteria of Anaplasmataceae family (such as *A. platys*, *E. canis*), *Mycoplasma* spp., *Rickettsia* spp., and *Bartonella* spp. using primer sets by conventional PCR (cPCR). The cPCR amplified targeted sequences of *Ehrlichia/Anaplasma* spp. (*16S rRNA* gene), *Babesia/Hepatozoon* spp. (*18S rRNA* gene), and *Rickettsia* spp. from the spotted fever group (*OmpA*). Positive samples for the genus of the stated species were then analyzed using primers that target species-specific sequences by cPCR or nested PCR. All primers and the target genes utilized for pathogen detection were outlined in Table 1. Pathogen-positive samples and distilled deionized water were utilized as the positive and negative controls, respectively, for all experiments. The PCR amplicons were examined using electrophoresis in a 1.5% agarose gel (LE agarose, Thermo Fisher Scientific, Waltham, MA, United States) and seen under a UV transilluminator (ATTO, Tokyo, Japan). DNA was extracted and purified from the positive amplicon by excising it from a gel using a Gel Extraction Kit from QIAGEN, Germany. The result, purified and having the desired sequences, underwent sequencing using the BigDye v3.1 Terminator Cycle Sequencing Kit in an ABI PRISM 3100 Genetic Analyzer by Applied Biosystem, United States.

The sequences’ chromatograms were evaluated with BioEdit software (version 7.5.2) and compared to sequences in the Genbank database using the Basic Local Alignment Search Tool (BLAST) on the U.S. National Center for Biotechnology Information (NCBI) website. This work evaluated the genetic variation of *Mycoplasma* spp. and *Rickettsia felis* by examining *16S rRNA* gene-based *Mycoplasma* spp. sequences (618 bp) and *gltA* gene-based *R. felis* sequences (654 bp) using phylogenetic analysis with the MEGA X tool. Phylogenetic trees were created using the maximum likelihood method and the most suitable substitution model. A bootstrap analysis with 1,000 replications was performed to evaluate the reliability of the branching patterns in the trees. Every sequence is provided with Genbank accession numbers, isolation sources, and countries of origin.

### 2.4 Statistical analysis

The OpenEpi application was utilized to calculate the detection rate of tick infestation, determine the percentage of discovered diseases, and estimate 95% confidence intervals (95% CI).^2^ A chi-square test was used to investigate the statistical relationship between the prevalence of tick infestation and the host independent factors. The odds ratio (OR) was calculated to assess the strength of the relationship between each category and tick infestation, determining if there was a significant association between them. The level of statistical significance was established at *p* ≤ 0.05. The study utilized a confidence level of 95%. The Statulator tool was utilized for data analysis.^3^

## 3 Results

### 3.1 Sample data and tick morphological identification

**Figure 2:**
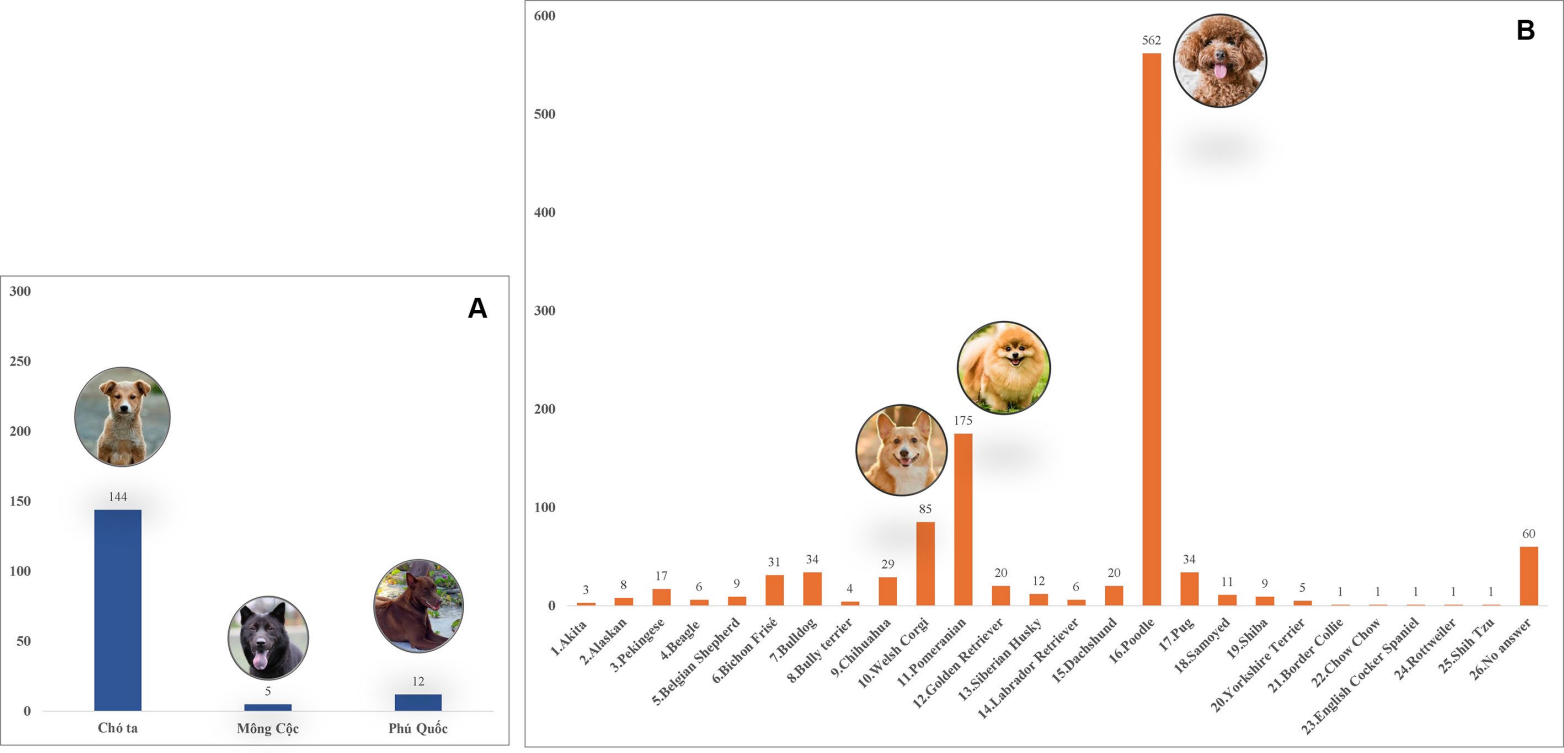
Different dog breeds surveyed in Vietnam during the study. **(A)** Domestic breeds. **(B)** Exotic breeds.

**Figure 3:**
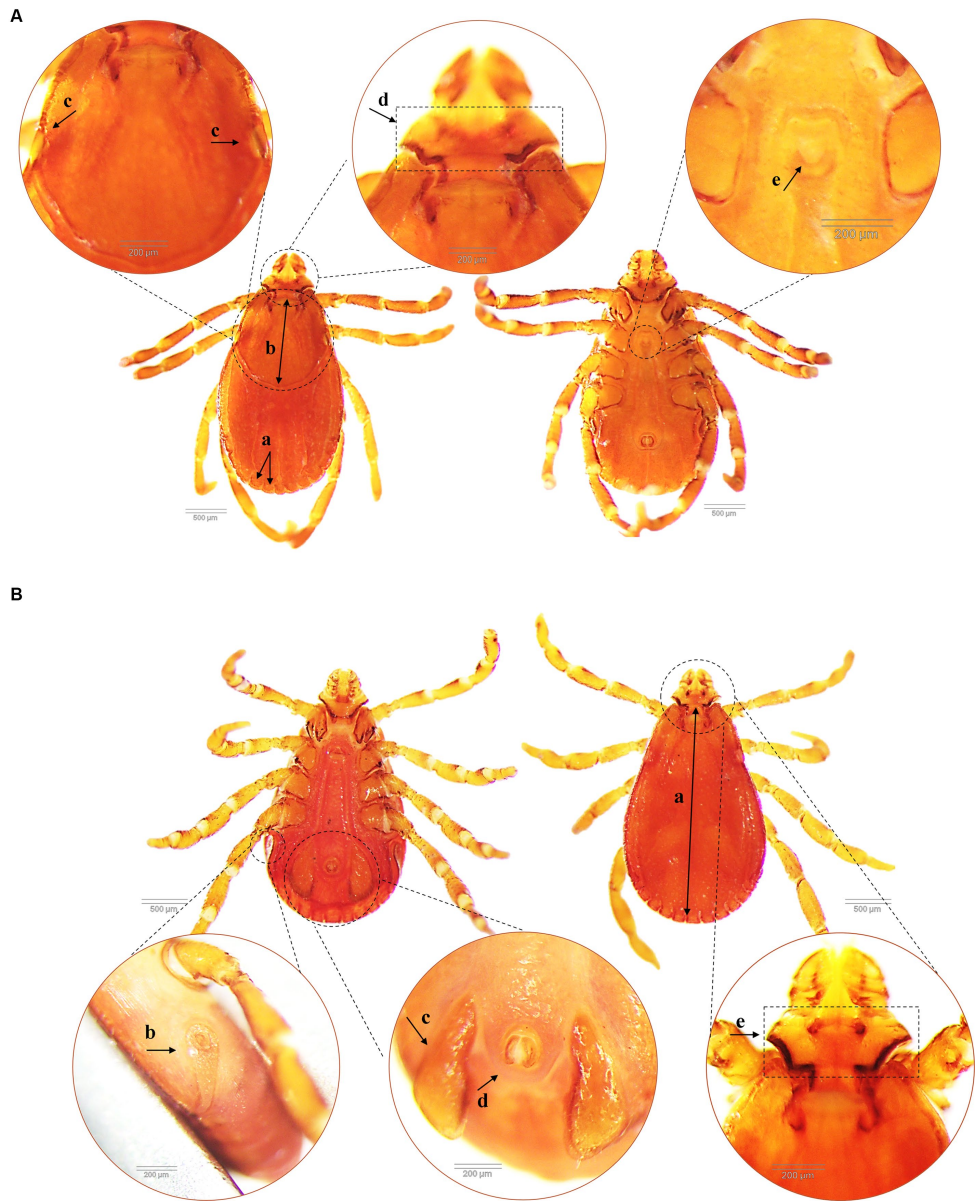
Morphological characteristics of *Rhipicephalus sanguineus* tick collected from dogs (dorsal and ventral side). **(A)** Female *R. sanguineus* with the festoon (similar for male) **(a)**, the scutum covers approximately 1/3 of the dorsal surface **(b)**, flat eyes **(c)**, hexagonal angulate basis capituli **(d)** and U-shaped genital aperture **(e)**. **(B)** Male *R. sanguineus* with the complete scutum covering most of the dosal surface **(a)**, coma-shaped spiracular plates (similar for female) **(b)**, subtriangular adanal plates **(c)**, anus groove posteriorly **(d)** and hexagonal angulate basis capituli **(e)**.

A total of 1,423 dogs participated in the survey, with 843 from Hanoi (59.24%), 424 from Ho Chi Minh City (29.8%), 132 from Nam Dinh (9.28%), and 24 from Dak-lak (1.68%; Figure 1). The enrolled dogs included: 630 females (44.27%), 729 males (51.23%), and 64 with unreported data (4.5%). The animals’ ages varied from 2 months to 20 years, with around 43.5% (619) of the population falling between one and 5 years old. The dogs were of more than 20 breeds, which were divided into three groups. Details of different breeds of dogs observed in the current study were presented in Figure 2; of which, 1,145 (80.46%) were of the exotic breed, 161 (11.31%) were of the domestic breed. The majority were indoor lifestyle (662, 46.52%), small size (≤3.5 kg; 462, 32.46%), and bathed at least once a week (707, 49.68%). Clinical symptoms such as skin and mucous membrane abnormalities were found to be statistically linked to the presence of tick infestation in dogs (Table A1). A total of 555 ticks were submitted for species identification and detection of microorganisms. In particular, based on the morphological typical traits, 33 larvae, 75 nymphs, and 447 adults (195 males and 252 females) are identified as belonging to the species complex *R. sanguineus* s.l. (Figure 3).

### 3.2 Geographical and tick seasonal distribution

**Figure 4:**
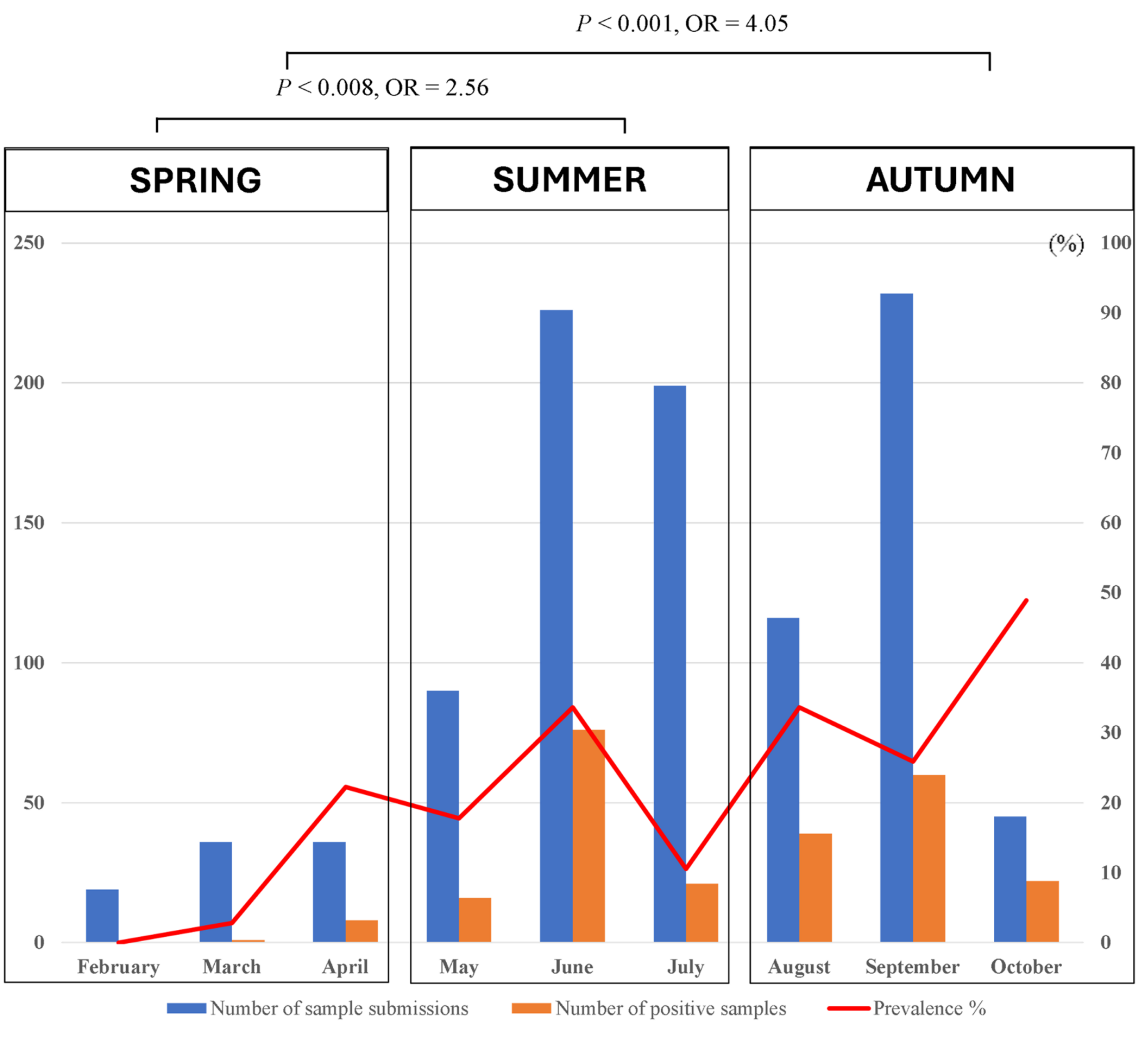
Monthly seasonal dynamics of the *Rhipicephalus sanguineus* tick found on owned dogs in northern Vietnam (2022).

Of 1,423 dogs examined, 414 (29.01, 95% CI: 26.79–31.51%) were carrying at least one tick at the time of sampling. According to statistical analysis, there was a significant difference determined between geographical areas and tick infestation rate in dogs. Tick infestation rate was highest in dogs sampled in Nam Dinh at 55.3% (73/132, 95% CI: 46.79–63.52) followed by Ho Chi Minh City at 39.86% (169/424, 95% CI: 35.31–44.59) and Hanoi 20.7% (170/843, 95% CI: 17.6–23.01). In Dak-Lak, tick presence was found in two dogs (8.33, 95% CI, 2.3–25.85). Besides, dogs residing in Nam Dinh and Ho Chi Minh City were 4.9 times (*p <* 0.001, 95% CI: 3.34–7.18%) and 2.62 times (*p <* 0.001, 95% CI: 2.03–3.39%) more likely to get tick infestation compared to those in Hanoi. Samples collected from the north of Vietnam were subjected to tick seasonal distribution analysis. During different seasons, more dogs were tick-infested from May to September and the number of tick-infested dogs declined between February and April (Figure 4). Additionally, the detection rate of tick infestation in dogs was statistically associated with the different seasons in the north. Specifically, the majority of tick-infested dogs in the study were detected in the warmer months of summer (21.94%, 113/515) and autumn (30.79%, 121/393) compared to spring (9.89%, 9/91). In summer and autumn, dogs had 2.56 (*p* = 0.008) and 4.05 times (*p* < 0.001) the odds of getting tick infestation compared to those in spring, respectively (Table 2).

**Table 2:** Risk factors associated with tick infestation in dogs in Vietnam.

Variables (*N* = 1,423)	No. tested dogs	No. tick-infested dogs	Df (χ^2^)*P*-value	OR (*P*-value)	95% CI
**Seasons**					
Spring (Feb/Mar/April)	91	9 (9.89)	2 (14.55) 0.0007	Ref	
Summer (May/June/July)	515	113 (21.94)		2.56 (0.008)	1.25–5.26
Autumn (Aug/Sep/Oct)	393	121 (30.79)		4.05 (0.001)	1.97–8.33
NG	424	171 (40.33)			
**Age (year)**					
Puppy (≤1)	560	123 (21.96)	2 (26.89) 0.0001	Ref	
Juvenile (1–5)	619	219 (35.38)		1.95 (0.001)	1.50–2.52
Adult (>5)	198	66 (33.33)		1.78 (0.001)	1.24–2.54
NG	46	6 (13.04)			
**Breed**					
Domestic	161	91 (56.52)	2 (63.64) 0.0001	3.71 (0.001)	2.65–5.21
Exotic	1,145	297 (25.94)		Ref	
Mix	74	20 (27.03)		1.06 (0.836)	0.62–1.80
NG	43	6 (13.95)			
**Sex**					
Female	630	184 (29.21)	1 (0.25) 0.617	0.94 (0.617)	0.75–1.19
Male	729	222 (30.45)		Ref	
NG	64	8 (12.50)			
**Body size (kg)**					
X-Small (≤3.5)	462	98 (21.21)	3 (44.22) 0.0001	Ref	
Small (3.6–7.5)	489	138 (28.22)		1.46 (0.012)	1.08–1.97
Medium (7.6–15)	267	114 (42.70)		2.77 (0.001)	1.99–3.85
Large (≥15)	131	52 (39.69)		2.44 (0.001)	1.61–3.70
NG	74	12 (16.22)			
**Life-style**					
Indoor	662	133 (20.09)	2 (173) 0.0001	Ref	
Outdoor	162	118 (72.84)		10.67 (0.001)	7.19–15.83
Semi-outdoor	558	162 (29.03)		1.63 (0.001)	1.25–2.12
NG	41	1 (2.44)			
**Bathing frequency**					
Every week	707	165 (23.34)	3 (30) 0.0001	Ref	
Every month	144	56 (38.89)		2.09 (0.001)	1.43–3.05
Every 2, 3 months	193	70 (36.27)		1.87 (0.001)	1.33–2.63
Once a year	320	116 (36.25)		1.87 (0.001)	1.40–2.49
NG	59	7 (11.86)			

NG, Not given; Df, Degrees of freedom; χ^2^, Chi-square test; OR, Odds ratio; CI, confidence intervals; Ref., reference used; No., number.

### 3.3 Risk factors associated with tick infestation in dogs in Vietnam

The relationship of tick infestation with different host attributes was statistically assessed in the present study and reported in Table 2 along with the number and percentage of tick infestation. Samples with missing information were excluded from the analysis. Majority of tick-infested dogs detected were juveniles (35.38%, 219/619), domestic breed (56.52%, 91/161), medium size (42.7%, 114/267), outdoor lifestyle (72.84%, 118/162) and bathed less than once a month (36.83%, 242/657), compared to the other groups of accordingly category. There was no statistically significant association between tick positivity and sex. The juveniles and adults had 1.95 times (*p* < 0.001) and 1.78 times (*p* < 0.001) the odds of getting tick infestation than the puppies, respectively. Domestic dogs were 3.71 times more likely to get tick infestation than exotic dogs (*p* = 0.001, 95% CI: 2.65–5.21). Dogs in small sizes were 2 times less likely to get tick infestation than those in medium (*p* = 0.001, 95% CI: 1.99–3.85) or large size (*p* = 0.001, 95% CI: 1.61–3.7). Dogs with outdoor lifestyle were significantly correlated with tick detection and had 10 times the risk of getting tick infestation compared to those living in-house in this study (*p* < 0.001, 95% CI: 7.19–15.83).

### 3.4 Microorganism detection in tick samples collected from dogs in Vietnam

Among the 177 tick pools that were examined, 146 (82.49%) had at least one pathogen found, with *Mycoplasma* spp. being the most common (78.53%, CI: 71.91–83.94), followed by *Anaplasma* spp. (37.29%, CI: 30.51–44.61), *R. felis* (5.08%, CI: 2.69–9.38), *B. vogeli*, and *H. canis* (2.82%, CI: 1.04–6.82). In 79 pools, a single pathogen was discovered (44.63%, CI: 37.5–51.99). Multiple pathogens were detected in 67 pools (37.85%, CI: 31.04–45.18; Table 3). In terms of tick stages, out of 151 adult-stage tick pools, 121 (80.13%) pools had *Mycoplasma* spp. infections. These were followed by pools infected with *Anaplasma* spp. (39.07%, 59/151), *R. felis* (5.3%, 8/151), *B. vogeli* (2.65%, 4/151), and *H. canis* (0.66%, 1/151). In this investigation, nymphs and adults were found to have the same infections, while larvae primarily had *Mycoplasma* spp. (87.5%; Table 4).

**Table 3:** The occurrence rate of pathogens in tick pools collected from dogs.

Pathogen detected (*N* = 177)	No. positive samples	Detection rate (%)	Confidence intervals 95%
**TBP total**	146	82.49	76.21–87.38
*Babesia vogeli*	5	2.82	1.04–6.82
*Hepatozoon canis*	5	2.82	1.04–6.82
*Mycoplasma* spp.	139	78.53	71.91–83.94
*Rickettsia felis*	9	5.08	2.69–9.38
*Anaplasma* spp.	66	37.29	30.51–44.61
**Single infection**	79	44.63	37.5–51.99
*Babesia vogeli*	2	1.13	0.31–4.02
*Mycoplasma* spp.	71	40.11	62.94–76.32
*Anaplasma* spp.	6	3.39	1.56–7.2
**Co-infection**	67	37.85	31.04–45.18
*B. vogeli + Mycoplasma* spp.	2	1.13	0.57–4.86
*Anaplasma* spp. *+ Mycoplasma* spp.	53	29.94	23.68–37.06
*Mycoplasma* spp. *+ R. felis*	5	2.82	1.92–7.93
*B. vogeli + Anaplasma* spp. *+ Mycoplasma* spp.	1	0.56	0.31–4.02
*H. canis + Anaplasma* spp. *+ Mycoplasma* spp.	3	1.69	0.57–4.86
*H. canis + Mycoplasma* spp. *+ R. felis*	1	0.56	0.31–4.02
*Anaplasma* spp. *+ Mycoplasma* spp. *+ R. felis*	2	1.13	0.57–4.86
*H. canis + Anaplasma* spp. *+ Mycoplasma* spp. *+ R. felis*	1	0.56	0.31–4.02

**Table 4:** Screening of pathogens in different life stages of *Rhipicephalus sanguineus* tick.

Stage of ticks	No. pools	No. positive pools for pathogens (%)	No. positive pools (%)				
			*B. vogeli*	*H. canis*	*Mycoplasma* spp.	*R. felis*	*Anaplasma* spp.
Adult	151	121 (80.13)	4 (2.65)	1 (0.66)	114 (75.5)	8 (5.3)	59 (39.07)
Nymph	18	18 (100)	1 (5.56)	4 (22.22)	18 (100)	1 (5.56)	7 (38.89)
Larva	8	7 (87.5)	0	0	7 (87.5)	0	0
Total	177	141 (79.66)	5 (2.82)	5 (2.82)	139 (78.53)	9 (5.08)	66 (37.92)

### 3.5 Sequencing identities and phylogenetic analysis

The nucleotide sequences for tick and each identified pathogen showed 99–100% similarity with sequences in the Genbank database. Specimens of *R. sanguineus* s.l. were genetically identified as part of the tropical lineage with 100% nucleotide identity (GenBank accession numbers MF425992–MF425994). The *18S rRNA* gene sequences of *B. vogeli* matched MT386936 with 100% identity, while *H. canis* showed 99.85% identity with MG050161. The *gltA* gene sequences of *R. felis* showed a high level of similarity, ranging from 99.84% to 100%, with sequences OM936910–MT019627. The *16S rRNA* gene sequences of *Mycoplasma* spp. demonstrated a nucleotide identity of 99.51% to 99.68% with *Mycoplasma wenyonii* sequences MF377464–MG948627. Whereas, *16S rRNA* gene sequences of *Anaplasma* spp. showed a high level of similarity, ranging from 99.03% to 99.67%, with sequences of the different species within the genus *Anaplasma* and *Ehrlichia* (EU090184–MN481611). However, none of the *Anaplasma/Ehrlichia* positive samples tested positive to *E. canis* or *A. platys* assays.

**Figure 5:**
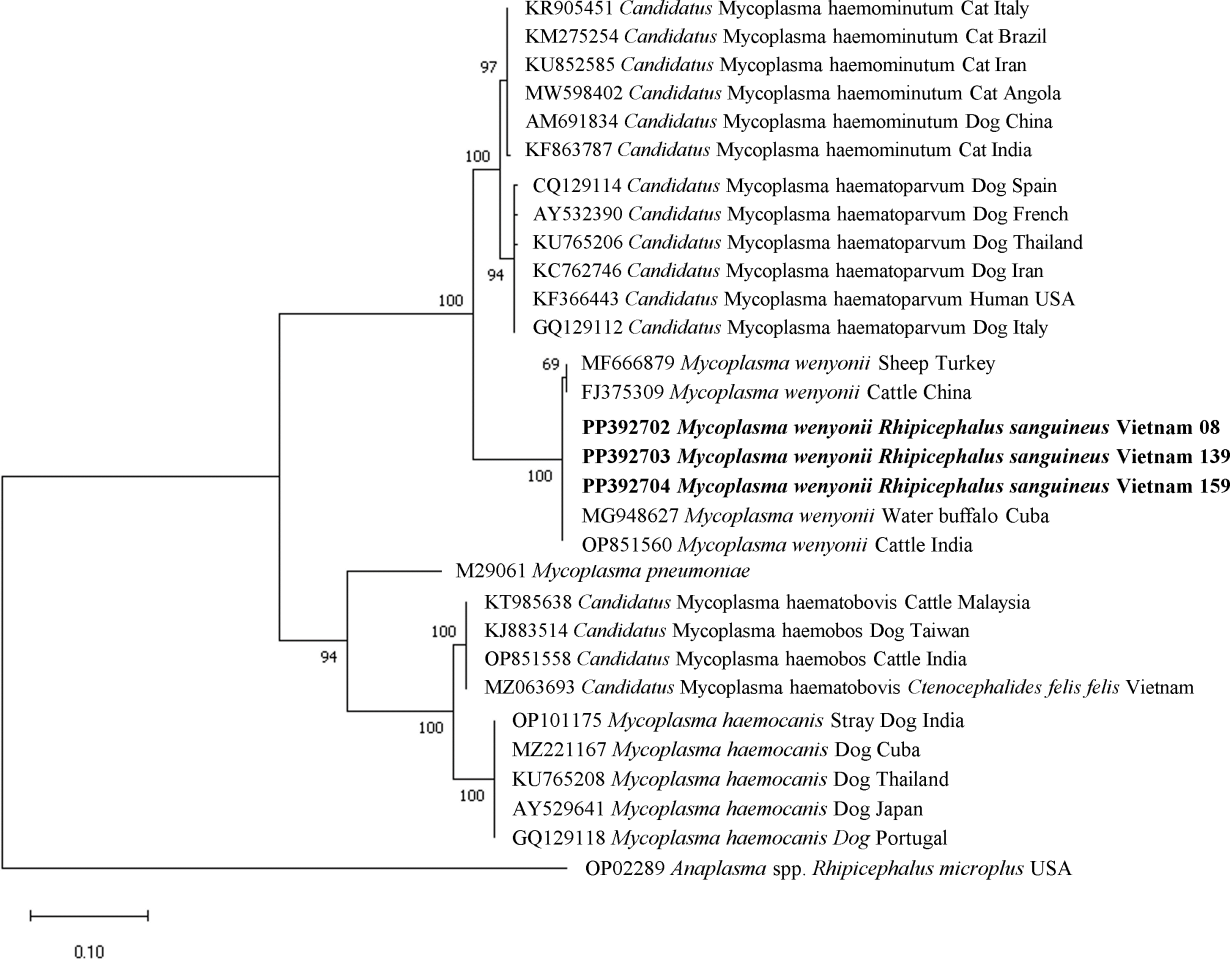
Phylogenetic analysis of *Mycoplasma wenyonii* based on the nucleotide sequences of a 618 bp fragment of *16S rRNA* gene using Tamura 2 parameter model. Isolates obtained from this study are presented in bold text.

**Figure 6:**
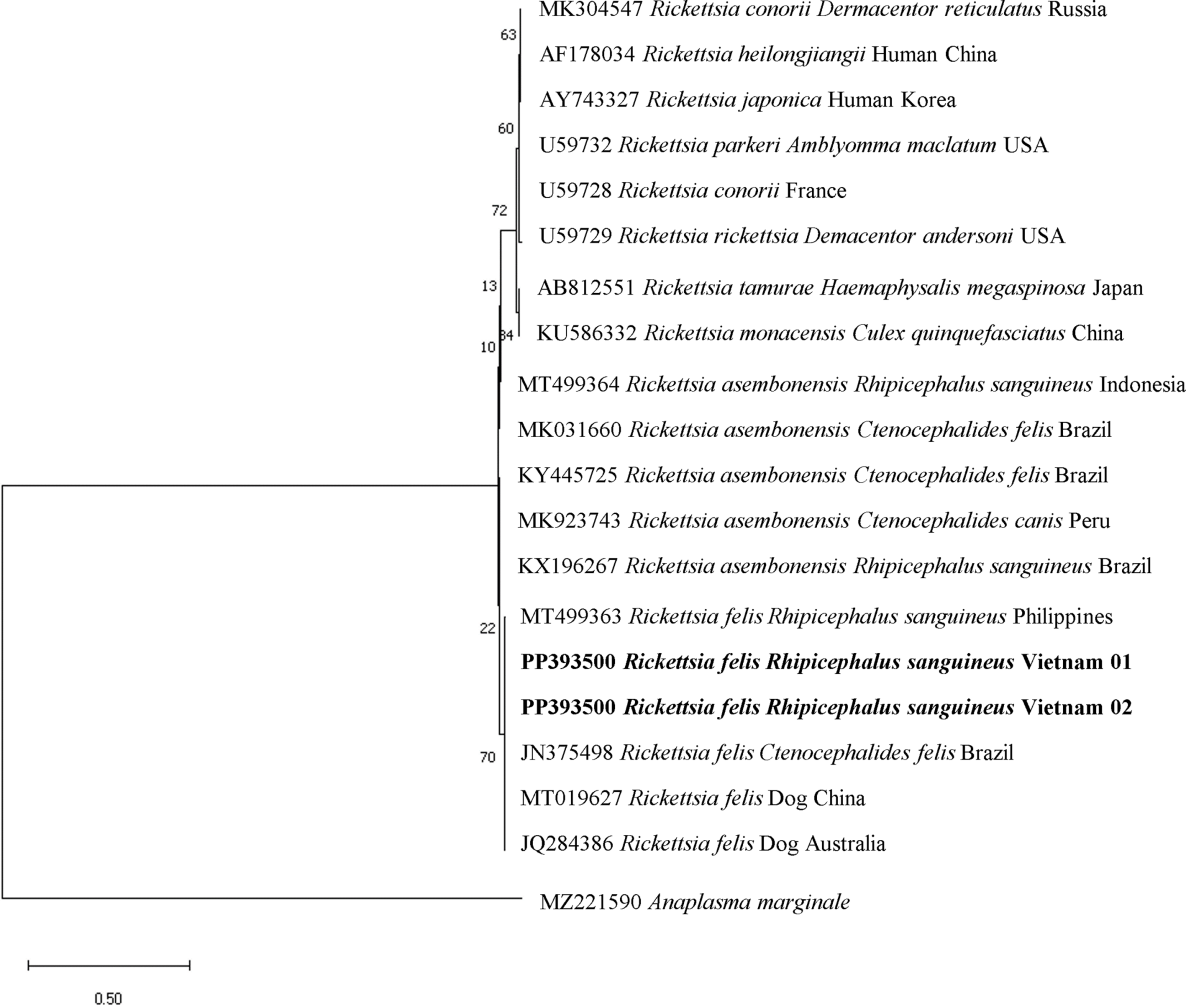
Phylogenetic analysis of *Rickettsia felis* based on the nucleotide sequences of a 654 bp fragment of *gltA* gene using Kimura 3 parameter model. Isolates obtained from this study are presented in bold text.

The molecular identification of particular sequences for *Mycoplasma* spp. and *R. felis* was confirmed by the clear separation of species-specific groups determined through phylogenetic analysis. The phylogenetic tree of the *16S rRNA* gene-based *Mycoplasma* spp. indicated that all *M. wenyonii* sequences (618 bp in length) grouped together in a distinct clade with strong support (99% bootstrap value). This clade included sequences from the same species found in various geographic locations, while excluding other *Mycoplasma* spp. species (Figure 5). Phylogenetic analysis of incomplete *gltA* gene sequences (654 bp in length) showed that all *R. felis* from dogs grouped together in a clade with reference sequences of the same species, supported by a bootstrap value of 70% (Figure 6).

The representative sequences produced in this research have been deposited in Genbank database with the following accession numbers: PP389595 and PP389596 for *R. sanguineus*, PP377903–PP377905 for *B. vogeli*, PP373785 for *H. canis*, PP392702–PP392704 for *M. wenyonii*, and PP393500–PP393501 for *R. felis.*

## 4 Discussion

The survey assessed the occurrence of ticks and the microorganisms they harbor on 1,423 dogs owned by clients, mainly in the rapidly expanding Vietnamese cities of Hanoi and Ho Chi Minh City, which house the majority of the country’s human and animal population, along with two other provinces (Nam Dinh and Dak Lak). The overall exposure to brown dog ticks was 29.01%. This study represents the initial systematic examination of tick distribution and frequency on privately owned dogs in Vietnam, utilizing voluntary enrollment in veterinarian care. The study analyzed seasonal tick dynamics and risk factors associated with tick infestation in the canines studied for the first time. The findings and data will provide valuable information to epidemiologists, veterinarians, and legislators to develop treatment and prevention measures for vectors and their related infections.

The tick species found in dogs in this study was *R. sanguineus* s.l. from the tropical lineage. Previous research in various regions of Vietnam has also confirmed that dogs are commonly infested by *R. sanguineus* s.l. (2, 8, 9). *Rhipicephalus sanguineus* is the predominant tick species that targets domestic animals in Southeast Asia (2). The study found that most dogs with ticks were taken to hospitals throughout the summer and autumn months in the Northern region, characterized by hot, humid weather and high precipitation (22). During these periods, ectoparasites such as ticks are more active due to climate conditions that support the survival and reproduction of blood-feeding arthropods. Previous research has indicated that *R. sanguineus* ticks prefer to lay their eggs at temperatures ranging from 20 to 30°C (23). Optimal parameters for nymph survival include a temperature of 20°C and a relative humidity of 85%. Engorged larvae and nymphs of brown dog ticks successfully underwent molting under various combinations of constant temperature ranging from 10 to 35°C and relative humidity from 15% to 95% (24). The higher incidence of tick infestation in dogs in Ho Chi Minh City compared to Hanoi may be due to the ticks being consistently active throughout the year because of the favorable climate in the region, with an average temperature ranging between 26°C and 29°C annually, resulting in a greater risk of dogs being exposed to ticks.

This study identified variations in the likelihood of tick infection in dogs based on age, breed, body size, lifestyle, and bathing frequency (Table 2). The lifestyle group was the primary variable related with tick infestations. The study found that dogs allowed to roam freely were more prone to tick infestation compared to those kept indoors. Dogs that roam outdoors freely are more likely to encounter ticks due to greater exposure to the environment and contact with tick-infested wild animals nearby. Studies undertaken in ASEAN nations have revealed tick incidence exceeding 80% in stray animals (25, 26). If the outdoor activity of dogs is the mechanism by which host-seeking tick activity is increasingly successful, then the pattern of association between rural areas and the risk of tick infestation is understandable in Vietnam; since dogs in rural regions reported often semi-domiciled and have free access to outside environment, predisposing them to ticks (27). Our research supported the claim that the rate of tick infestation in dogs in Nam Dinh, a rural location, was notably greater than in Hanoi and Ho Chi Minh City, metropolitan areas. Tick infestation is notably higher in domestic dogs than in exotic breeds. The various reasons for owning a dog may have been the cause. Most domestic breed dogs in Vietnam are typically raised outdoors for guarding purposes and are kept in kennels, making them more susceptible to tick infestations compared to exotic breeds that are usually kept as pets for companionship and may even share a bed with their owners. Older dogs, including juveniles and adults, are more susceptible to tick infestation in this study, maybe because they spend more time outdoors than pups. Small dogs who are bathed frequently may have a reduced chance of tick attachment, according to the study. Small dogs may be easier to bathe than larger dogs due to their size, making them more approachable and manageable. Previous research on the relationship between tick infestation and gender found that male dogs had a much higher risk of infestation (28); however, this relationship was not seen in the current study.

The molecular testing revealed that the majority of tick samples were infected with *Mycoplasma* spp. (78.53%) rather than *Anaplasma* spp., *Ehrlichia* spp., *H. canis*, *B. vogeli*, or *R. felis*. The transmission pathways of hemoplasmas in dogs are under discussion, with blood-feeding arthropods including fleas and ticks proposed as potential vectors (29). Mycoplasmosis is not considered to be a tick-borne pathogen, however some hemotrophic mycoplasms that are significant in veterinary and public health can sometimes be found in ticks and co-infect hosts with established tick-borne pathogens. *Mycoplasma* spp. were found alongside other microorganisms, including *Anaplasma* spp., *B. vogeli*, *H. canis*, and *R. felis* in tick samples collected from the same host. The findings suggest that vertebrate hosts can be infected with several tick-borne diseases either by a single tick carrying multiple pathogens or by distinct ticks carrying individual pathogens. Hemotropic mycoplasma species, specifically *Mycoplasma haemocanis* and *Candidatus* Mycoplasma haematoparvum, have been recently identified in ticks and dogs in Vietnam and various regions of Southeast Asia, causing canine mycoplasmosis (3, 30, 31). A significant detection rate of *M. wenyonii* was unexpectedly detected in brown dog ticks and all three different stages of ticks (adults, nymph, larva) in this study, despite it being regularly reported to infect cattle and sheep after phylogenetic analysis. The role of ticks in the epidemiology of this pathogen remains uncertain.

The second most prevalent bacterium detected in tick samples in this study was *Ehrlichia/Anaplasma* spp. (37.29%). *Anaplasma platys* was the species found in dogs in Vietnam (30) and *E. canis* commonly detected in ticks and canines in different countries in Southeast Asia such as Thailand (3), Cambodia (32), or Philippines (33). However, all tick pools in the current study tested negative for *A. platys* and *E. canis,* when further analyzed for *A. platys* and *E. canis* identification using species-specific primers. These findings indicate other species belonging to the genus *Ehrlichia* and *Anaplasma* might be circulating in ticks in the studied areas. A further investigation on this bacteria in ticks with an increased number of samples is suggested to identify and investigate the phylogeny of these bacteria in Vietnam. *Rickettsia felis* is an emerging insect-borne rickettsial pathogen causing flea-borne spotted fever, which can be found in mammalian hosts and arthropods worldwide (34), and *Ctenocephalides felis* fleas—known as the main vector and reservoir for this pathogen (35). This pathogen has been recently detected in *C. felis* from dogs and cats in several parts of Vietnam (36–38). Our study found *R. felis* for the first time in both adult and nymph stages of *R. sanguineus* from owned dogs in Vietnam. The discovery of *R. felis* in both adult and nymph stages of *R. sanguineus*, as well as the high frequency of this tick species parasitizing dogs in the study, raises concerns about the potential transmission of *R. felis* to residents in the areas under investigation. Human cases of febrile sickness caused by *R. felis* have been reported in Thailand (39), Indonesia (40), and Laos (41). In Vietnam, *R. felis* was recently discovered and detected in humans by a molecular assay (42). The agent was reported to cause a severe undifferentiated fever in patients who displayed an eschar, a common clinical indicator that sometimes appeared at the location of the tick or mite bite (43, 44). *Rhipicephalus sanguineus* can transmit other species of the spotted fever group Rickettsiae, including *Rickettsia rickettsii*, which causes Rocky Mountain spotted fever, a severe and potentially fatal tick-borne disease; however, *R. rickettsii* has not been reported yet in Southeast Asia (43). The presence of *R. felis* and other pathogens found in *R. sanguineus* in this study suggests that these pathogens may also be present in the canines under investigation, posing a risk of infection to the host. This emphasizes the significance of performing epidemiological investigations on tick-borne infections in canines in the future. Moreover, *H. canis* and *B. vogeli* were discovered in a substantially lower percentage, which showed similar to the previous studies (9, 38). In summary, the identification of the above mentioned pathogens in ticks highlights the potential of this ectoparasite to transmit harmful organisms, posing a threat to both human and animal health (1, 4).

## 5 Conclusion

The study found that the brown dog tick (*R. sanguineus* s.l.) is common in dogs in the studied areas of Vietnam. This study validated multiple parameters that contribute to the probability of tick infestation in dogs, essential for successful veterinary therapies. The data indicate the presence of *R. felis*, a causative agent of spotted fever, in *R. sanguineus* ticks in Vietnam for the first time, which poses substantial health concerns to animals.

## Data availability statement

The datasets presented in this study can be found in online repositories. The names of the repository/repositories and accession number(s) can be found in the article/supplementary material.

## Ethics statement

The animal studies were approved by the Ethics Committee of Obihiro University of Agriculture and Veterinary Medicine accepted the protocol for using animal samples in this work (Permit for animal experiment: 21-25; DNA experiment: 1725-5). The studies were conducted in accordance with the local legislation and institutional requirements. Written informed consent was obtained from the owners for the participation of their animals in this study.

## Author contributions

TD: Conceptualization, Data curation, Formal analysis, Investigation, Methodology, Project administration, Software, Visualization, Writing – original draft, Writing – review & editing. LKB: Funding acquisition, Resources, Writing – original draft, Writing – review & editing. RU-S: Methodology, Writing – original draft, Writing – review & editing. TI: Methodology, Writing – original draft, Writing – review & editing. TH: Methodology, Writing – original draft, Writing – review & editing. IZ: Methodology, Writing – original draft, Writing – review & editing. ZM: Methodology, Writing – original draft, Writing – review & editing. LH: Methodology, Writing – original draft, Writing – review & editing. UKM: Methodology, Writing – original draft, Writing – review & editing. MA: Methodology, Writing – original draft, Writing – review & editing. SAE-S: Methodology, Writing – original draft, Writing – review & editing. XX: Conceptualization, Funding acquisition, Project administration, Resources, Supervision, Validation, Writing – original draft, Writing – review & editing. KK: Funding acquisition, Writing – review & editing.

## Footnotes

^1^https://climateknowledgeportal.worldbank.org/country/vietnam/climate-data-historical (Accessed 22 February 2024).

^2^ https://www.openepi.com/Proportion/Proportion.htm

^3^ https://statulator.com/stat/chisq.html

## Appendix

**Table A1:** Clinical signs associated with the detection of tick infestation in dogs.

Clinical signs	Tested samples	Tick-infested samples (%)	Df (χ^2^)*p*-value	OR (*p*-value)	CI 95%
**Mucus membrane**					
Normal	1,285	343 (26.69)	3 (97.14) 0.0001	Ref	
Jaundice	14	14 (100)		Invalid	
Pale	68	49 (72.06)		7.08 (0.001)	4.11–12.20
Hemorrhage	3	1 (33.33)		Invalid	
NG	53	7 (13.21)			
**Skin and hair status**					
Normal	1,120	262 (23.39)	1 (118.62) 0.001	Ref	
Abnormal	251	146 (58.17)		4.55 (0.001)	3.42–6.06
NG	52	6 (11.54)			

NG, Not given; Df, Degrees of freedom; χ^2^, Chi-square test; OR, Odds ratio; CI, confidence intervals; Ref., reference used; No., number.
